# Identification and Genomic Analyses of a Multidrug Resistant Avian Pathogenic *Escherichia coli* Coharboring *mcr-1*, *bla*_TEM-176_ and *bla*_CTX-M-14_ Genes

**DOI:** 10.1155/2024/9332418

**Published:** 2024-02-16

**Authors:** Zhiyang Wang, Xinyu Wang, Weiqi Guo, Di Wang, Jiangang Hu, Beibei Zhang, Jingjing Qi, Mingxing Tian, Yanqing Bao, Haihua Li, Shaohui Wang

**Affiliations:** ^1^Shanghai Veterinary Research Institute, Chinese Academy of Agricultural Sciences, Shanghai 200241, China; ^2^Tianjin Key Laboratory of Agricultural Animal Breeding and Healthy Husbandry, College of Animal Science and Veterinary Medicine, Tianjin Agricultural University, Tianjin 300384, China

## Abstract

The emergence and transmission of the colistin-resistance gene *mcr* and extended-spectrum *β*-lactamase (ESBL) encoding genes pose a significant threat to global public health. In recent years, it has been reported that *mcr-1* and ESBL genes can coexist in single bacteria strain. The objective of this study was to characterize a multidrug-resistant (MDR) avian pathogenic *Escherichia coli* (APEC) isolate carrying *mcr* and ESBL encoding genes in China. A total of 200 APEC isolates were collected for antimicrobial susceptibility testing by Kirby–Bauer (K–B) disk method. The MDR strain EC012 were then further analyzed for minimum inhibitory concentrations, antimicrobials resistance genes (ARGs) detection, conjugation, and whole-genome sequencing (WGS). Among all APEC isolates determined by K–B disk method, strain EC012 was resistant to almost all the antimicrobials, including polymyxin B, cefotaxime, and ceftazidime. Moreover, EC012 harbored ARGs *mcr-1*, *bla*_TEM-176_, and *bla*_CTX-M-14_. WGS analysis revealed that EC012 belonged to epidemic APEC serotype O1:H16 and multilocus sequence type ST295. EC012 consisted of one chromosome and six plasmids, encoding a broad ARGs. The *bla*_CTX-M-14_, *mcr-1* or *bla*_TEM-176_ genes were located on conjugative plasmids pEC012-1 or pEC012-5, respectively. These plasmids were successfully transferred to transconjugants and resulted in the resistance to polymyxin B, cefotaxime, and ceftazidime. This study indicated that APEC was a potential reservoir of colistin-resistance gene *mcr-1* and ESBL encoding genes, and highlighted the necessity for enhanced monitoring of ARGs dissemination among bacteria from different origins.

## 1. Introduction

Avian pathogenic *Escherichia coli* (APEC) is a kind of extraintestinal pathogenic *E. coli* (ExPEC), which can cause avian colibacillosis and lead to worldwide economic losses in the poultry industry [1]. The antimicrobials were used to treat APEC infection, however, the inappropriate use of antibiotics accelerated to the emergence of multidrug-resistant (MDR) bacteria. Previous studies revealed that APEC strains showed MDR and to be important reservoirs for antimicrobials resistance genes (ARGs), suggesting its potential risk for humans [2].

Colistin is a cationic amphiphilic lipopeptide antibacterial agent. As a last resort antimicrobial, colistin is an important antimicrobial for human treatment of Carbapenem-resistant Enterobacteriaceae (CRE) infections [3]. However, it has been widely used in livestock production, especially in pigs, for a long time [4]. With the global spread of colistin resistance gene *mcr*, a transferable plasmid-mediated colistin resistance gene encoding for phosphoethanolamine transferase, the clinical application of colistin is under serious threat [5].

Currently, multidrug resistance in *E. coli* is a major concern, especially resistance exhibited by extended-spectrum *β*-lactamase (ESBL)-producing strains to third- or fourth-generation cephalosporins, fluoroquinolones, aminoglycosides, tetracyclines, and trimethoprim–sulfamethoxazole [6, 7]. Liu et al. [8] indicated a plasmid-mediated colistin-resistance gene *mcr-1* in *E. coli*, isolated from humans and livestock in China, which could be transferred among different bacteria. The rapid spread and evolution of the mobile colistin-resistance gene *mcr* has become a global concern. To date, *mcr-1*–*mcr-10* have been widely identified around the world [9], and new genotypes are still being discovered. Since 2016, WHO has listed colistin as one of the crucially important antimicrobials [10]. Many countries have approved the withdrawal of colistin as a feed additive in animals [4, 11].

The gene *mcr-1* is commonly carried by plasmids of various replicon types, among which IncHI2 is relatively common [12]. Similarly, many conjugative plasmids, such as IncF, IncI, IncK, and IncHI2, play important roles in the global dissemination of ESBL genes [12]. The most common ESBL-encoding genes are *bla*_CTX-M_, *bla*_TEM_, *bla*_SHV_, and *bla*_OXA_ [13]. Insertion sequences (IS) were also involved in the mobilization of *bla*_CTX-M-type_ and *bla*_TEM_ genes via transposition or homologous recombination [14]. It was reported the horizontal transfer of conjugative plasmid is a crucial contributor to ARGs spread [15].

Recently, the resistance genes *bla*_CTX-M-type_, *bla*_TEM_, and *mcr-1* were detected simultaneously in Enterobacteriaceae isolated from humans, food products, and animals [16]. A high prevalence of *bla*_CTX-M-type_, *bla*_TEM_, and *mcr-1* in *E. coli* was found in both human and animal samples [4, 17]. It implies that a single pathogenic bacterial strain that carries a large number of ARGs could be a serious public health threat. In this study, the genomic features of a MDR APEC strain cocarrying the *mcr-1*, *bla*_CTX-M-14_, and *bla*_TEM-176_ genes in China was characterized.

## 2. Materials and Methods

### 2.1. Bacteria Collection

A total of 200 APEC isolates were used for the MDR bacteria screening. These APEC strains were isolated from diseased ducks or chicken with avian colibacillosis in Jiangsu, Anhui, Fujian, and Shandong provinces of China by our previous studies [4, 18,].

### 2.2. Antimicrobial Susceptibility Testing

The antimicrobial susceptibility of APEC strains was determined using Kirby–Bauer (K–B) disk method according to the protocol of Clinical and Laboratory Standards Institute (CLSI) [19, 20]. A total of eight different antimicrobials classes were tested, including *β*-lactams (ampicillin, amoxicillin, ceftriaxone, cephalothin, cefotaxime, and cefuroxime), aminoglycosides (streptomycin and kanamycin), quinolones (ciprofloxacin and enrofloxacin), sulfonamides (trimethoprim/sulfamethoxazole), phenicol (chloramphenicol and florfenicol), tetracycline (tetracycline and tigecycline), colistin (polymyxin B), and carbapenems (imipenem, meropenem, and ertapenem). APEC strain EC012 displayed resistance to almost all of the tested antimicrobials. Thus, the minimum inhibitory concentrations (MICs) of polymyxin B, cephalosporins, carbapenems, rifampicin, tigecycline, and tetracycline of strain EC012 were determined using a broth microdilution method recommended by the joint European Committee on Antimicrobial Susceptibility Testing (EUCAST) and CLSI guidelines [20]. The antimicrobial agents tested in this study were provided by Wenzhou Cont Biotechnology Co., Ltd., China. *E. coli* ATCC 25922 was used as a quality control strain.

### 2.3. Detection of Antimicrobials Resistance Genes

The APEC strains were detected for the presence of ARGs (including *mcr-1*, *bla*_CTX-M-9G_, and *bla*_TEM_) by PCR, using published primer sequences [8, 21]. Positive controls and negative controls were performed during the PCR amplification. The PCR amplicons were purified and sequenced to confirm the ARGs.

### 2.4. Whole Genome Sequencing and Analysis

Total DNA was extracted with the QIAmp DNA Mini Kit (Qiagen GmbH, Qiagen Strasse 1, Hilden, Germany) according to the manufacturer's instructions. The genomic DNA was subjected to whole genome sequencing (WGS) using Illumina NovaSeq with paired-end reads of 150 bp length and the Oxford Nanopore platforms. Sequencing reads including short-read and long-read data were assembled with CANU v1.7.1 and HGAP v4 with the hybrid strategy [22]. The plasmid sequences were initially annotated using the RAST server (http://rast.nmpdr.org) and corrected manually.

Multilocus sequence type (MLST) was determined by analyzing the sequences of seven house-keeping genes using mlst.2 [23]. A maximum-likelihood tree based on single nucleotide polymorphism (SNP) differences of the 103 *E*. *coli* genomes (all belongs to ST295) was generated using EnteroBase [24]. Serotyping was performed using the SerotypeFinder 1.2 web tool [25]. The plasmid replicon genotypes were identified using PlasmidFinder 2.1 (https://cge.food.dtu.dk/services/PlasmidFinder/). *OriT*finder was used to find the origin of transfer (*oriT*), relaxase, T4SS gene clusters, and the type IV coupling protein (T4CP; *oriT*finder (https://tool-mml.sjtu.edu.cn/oriTfinder/oriTfinder.html)). IS elements were identified using ISfinder (https://isfinder.biotoul.fr/). Besides, the CARD-RGI online tool (https://card.mcmaster.ca/analyze/rgi) was used to search for ARGs and determine point mutations. The comparative analysis and plasmid maps were generated using Easyfig 2.2.5 and BLAST Ring Image Generator (BRIG) -0.95-dist.

### 2.5. Conjugation Assay

The transferability of ARGs and plasmid(s) from the donor strains APEC into recipient strain *E*. *coli* 600 was tested by a filter-mating conjugation experiment. Transconjugants were select on LB agar with rifampicin (500 *μ*g/mL), cefotaxime (4 *μ*g/mL), and polymyxin B (2 *μ*g/mL) or rifampicin (500 *μ*g/mL), cefotaxime (4 *μ*g/mL), and ciprofloxacin (2 *μ*g/mL). Colonies that grew on these selective plates were further confirmed by antimicrobial susceptibility tests. To demonstrate transferability of *mcr-1*, *bla*_CTX-M-14_, and *bla*_TEM-176_ genes along with the conjugative plasmid, transconjugants were PCR amplified.

### 2.6. Nucleotide Sequence Accession Numbers

The complete nucleotide sequences of six plasmids and chromosome in EC012 isolate were submitted to GenBank with the following accession numbers: chr-EC012 (CP119577.1), pEC012-1 (CP119578.1), pEC012-2 (CP119579.2), pEC012-3 (CP119580.2), pEC012-4 (CP119581.2), pEC012-5 (CP119582.2), and pEC012-6 (CP119583.2).

## 3. Results

### 3.1. Antimicrobial Susceptibility of APEC Isolates

These APEC isolates showed different antimicrobial resistance profiles, and all the isolates were resistant to at least three different classes of antimicrobials, especially showed highly resistant to quinolones, tetracycline, and sulfonamides. APEC isolates showed moderate resistance to phenicol and aminoglycosides. Among them, APEC EC012 was resistant to most of the tested antimicrobials, including aminoglycosides, quinolones, sulfonamides, chloramphenicol, and tetracyclines. Moreover, EC012 were coresistant to *β*-lactams and polymyxin B, including cefotaxime (>64 mg/L), cefuroxime (>32 mg/L), ceftriaxone (>64 mg/L), cefazolin (>64 mg/L), and polymyxin B (4 mg/L). However, this isolate was susceptible to carbapenem (meropenem and imipenem) and tigecycline (Table 1). PCR detection showed that EC012 carried ARGs *mcr-1*, *bla*_CTX-M-9G_, and *bla*_TEM_.

### 3.2. Whole Genome Analysis

**Figure 1:**
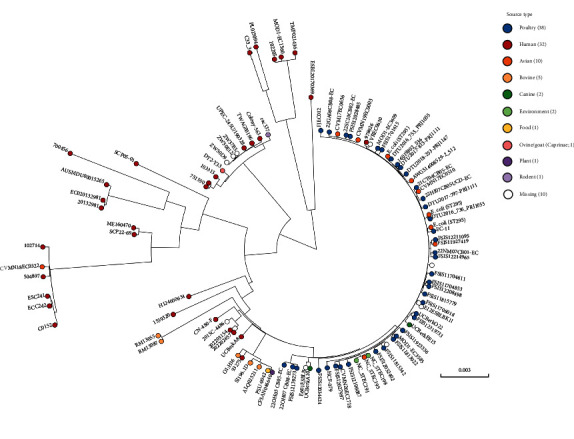
Phylogenetic tree of ST295 *E. coli* isolates based on core genome single nucleotide polymorphisms. The tree is based on 103 genomes and rooted at midpoint. Classification according to source is colored according to the figure's scheme.

**Figure 2:**
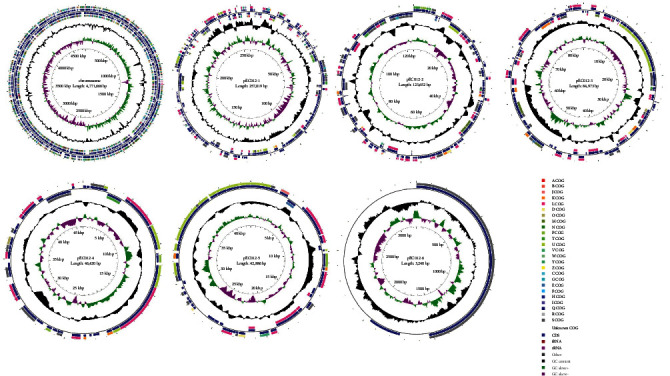
Structure of APEC EC012 chromosome and plasmids.

The genome size of EC012 was 4,771,666 bp with GC content of 50.76%. Based on in silico EcOH typing and MLST, EC012 strain belonged to serotype O1:H16 and sequence type ST295, one of the epidemic serotypes and STs in APEC. The SNP analysis was performed on the 103 ST295 *E. coli* genomes, and the phylogenetic tree revealed that EC012 clustered with *E*. *coli* isolates from poultry and human (Figure 1). According to the Plasmidfinder, there were at least six replication subtypes in EC012, including IncHI2/HI2A/N (pEC012-1), IncN/FIA (pEC012-2), IncFIB (pEC012-3), IncFII (pEC012-4), IncX1_1 (pEC012-5), and ColRNAI (pEC012-6) (Figure 2).

Whole genome sequencing indicated that the quinolone resistance genes *gyrA* mutant (D87N and S83L) and *parC* mutant (S80I), as well as the fosfomycin resistance gene *glpT* mutant (E448K) were located in the chromosome. Whereas, genes *mcr-1*, *qnrS*, *aadA2*, *cmlA1*, *sul3*, and *bla*_CTX-M-14_ were located in plasmid pEC012-1, *bla*_TEM-176_, *qnrS1*, *floR*, and *aph(3′)-Ia* were located in plasmid pEC012-5. However, no ARG was found in other four plasmids.

### 3.3. Characterization of a Novel Hybrid *mcr-1*-Bearing Plasmid pEC012-1

The *bla*_CTX-M-14_ and *mcr-1 co*bearing plasmid pEC012-1 was 257,014 bp in size and had an average guanine and cytosine (GC) content of 46.46%. The results of annotation indicated that pEC012-1 harbored 294 open reading frames (ORFs) and was a hybrid plasmid containing multiple replicons including IncHI2, IncHI2A, and IncN. CARD analysis identified a number of ARGs in pEC012-1, including aminoglycosides resistance genes *aac(3)-IVa*, *aph(3′)-Ia*, *aph(4)-Ia*, *aadA1*, and *aadA2*; macrolides resistance gene *mph(A*); *β*-lactamases resistance gene *bla*_CTX-M-14;_ phenicols resistance genes *cmlA1* and *floR*; quinolones resistance gene *qacH*; tetracyclines resistance gene *tet(M*),; fosfomycin resistance gene *fosA3*,; and sulfonamides resistance genes *sul2* and *sul3* in addition to colistin resistance gene *mcr-1* (Table 2).

**Figure 3:**
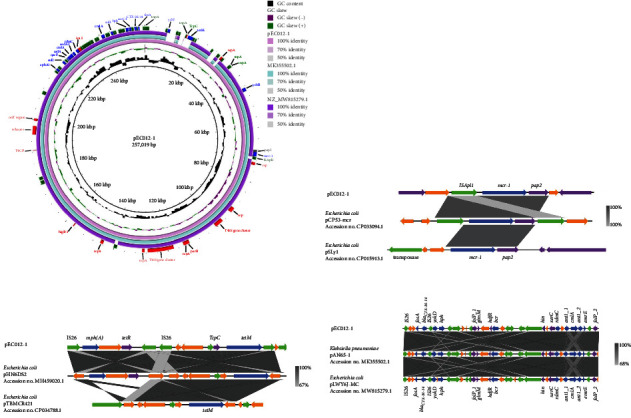
Comparative sequence analysis of *bla*_CTX-M-14_ and *mcr-1* carrying plasmids from *E. coli* isolates and genetic features of pEC012-1. (a) Comparison of *mcr-1*-harbouring IncHI2/HI2A/N plasmid pEC012-1 with MG656414.1 and MW815279.1. (b) Comparison of the *mcr-1*-haboring homologous fragment structure. (c) Comparison of the *mph(A)*-harboring homologous fragment structure. (d) Comparison of the *bla*_CTX-M-14_-haboring homologous fragment structure. Green color indicates insertion sequences and transposase, blue color indicates antimicrobials resistance genes, red color indicates backbone of plasmid, and purple color indicates specific protein.

Interestingly, these resistance genes were distributed in three distinct regions of pEC012-1, a 3,539 bp *mcr-1*-containing cassette IS*Apl1*-*mcr-1*-*pap2*, a 12,032 bp *tet(M*)-harboring segment organized as IS*1*-IS*6*-*tet(M*)-*tcpC*-Tn*3*, *partial*-MFS transporter-*mph(A*)-IS*26* were located in the backbone, and a 14,864 bp *bla*_CTX-M-14_-haboring segment including the remaining resistance genes which were dispersed between multiple ISs. The comparative analysis showed that pEC012-1 aligned well with MG656414.1 and MW815279.1, but MG656414.1 and MW815279.1 lacked the 12,032 bp *tet(M*)-harboring segment. Additionally, genes responsible for plasmid maintenance including *parB* and *higB* (toxin–antitoxin system), conjugation, and transfer (*tra*-relative genes) were also observed in the backbone of pEC012-1 (Figure 3).

### 3.4. Characterization of a *bla*_TEM-176_-Bearing IncX1 Plasmid pEC012-5

**Figure 4:**
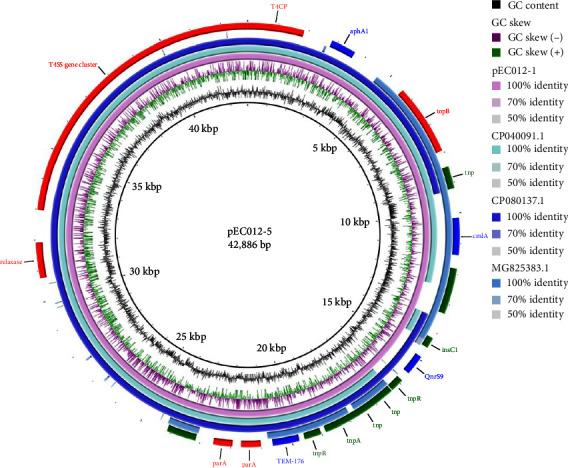
Comparative sequence analysis of *bla*_TEM-176_ bearing plasmid from *E. coli* isolates. Comparison of *bla*_TEM-176_-harbouring IncX1 plasmid pEC012-5 with CP040091.1, CP080137.1, and MG825383.1. Green color indicates insertion sequences and transposase, blue color indicates antimicrobials resistance genes, and red color indicates backbone of plasmid.

The *bla*_TEM-176_-bearing plasmid pEC012-5 (42,886 bp) was found to contain 51 ORFs with a GC content of 45.73% (Figure 4). The plasmid pEC012-5 belonged to the IncX1_1 incompatibility group. Intriguingly, the comparative analysis showed that the MDR region was similar to pFT130-1 (Genbank accession no: CP040091.1), p1079-IncFIB-N (Genbank accession no: MG825383.1), and pPK8277-49kb (Genbank accession no: CP080137.1) from *E. coli*. Besides of lacking *aphA1* or *cmlA1*, above *bla*_TEM-176_ was a fragment of IS*6* instead of Tn*2* (Figure 4).

Sequence analysis by *oriT*finder identified the type IV coupling protein (T4CP) gene, relaxase, and the T4SS gene cluster on plasmid pEC012-5, but *oriT* was absent (Figure 4). Given that the four components are necessary for conjugation in self-transmissible plasmids [26], indicating there were possible mutations of the *oriT* gene.

### 3.5. Transferability of Plasmids

In conjugation experiments, two plasmids (pEC012-1 and pEC012-5) could transfer from APEC EC012 into the recipient *E. coli* 600. The two transconjugants had similar antimicrobials profiles as parental clinical APEC isolate. In transconjugant *E. coli* 600 with pEC012-1, the *mcr-1* and *bla*_CTX-M-14_ genes were successfully transferred into the recipient *E. coli* 600 strain along the conjugative plasmid via transfer frequencies ranged from 10^−3^ to 10^−4^. The MIC values for polymyxin B in the transconjugant was 2 *μ*g/mL, which was higher than the original MIC of the *E. coli* 600 strain (Table 1). The MIC values for cefotaxime, cefuroxime, and ceftriaxone in the transconjugants were much higher than the original MIC of the *E. coli* 600 strain (Table 1). In transconjugant *E. coli* 600 with pEC012-5, the *bla*_TEM-176_ was successfully transferred to the recipient *E. coli* 600 strain as well. The MIC values for ceftriaxone is over eightfold than *E. coli* 600 and twofold than *E. coli* 600 with pEC012-1, as well as the MIC value of ciprofloxacin was thirty-two-fold higher than *E. coli* 600.

## 4. Discussion

As the last line defense against MDR *E. coli* infections, colistin has been widely used to treat infections caused by CRE [3]. Additionally, as the poultry and swine industries accounted for 96% of total colistin sulfate livestock use [4], the presence of *mcr* in them provides evidence that colistin treatment has promoted the transmission of *mcr*, with livestock as the primary reservoir. Colistin-resistant *E. coli* can then spread by contaminating animal-derived food or contaminating crops by excrement to threaten public safety [5].

The WGS analysis indicated that *mcr-1* was located on IncHI2/HI2A/N hybrid plasmid. IncHI2 plasmids were first reported in *Serratia marcescens* in the United States in 1969 and were recovered from environmental and human *Salmonella enterica* serovar Panama in Chile in the 1980s [27]. A lot of evidence suggests that *mcr-1* was located on a wide range of conjugative plasmids, IncI2, IncHI2, IncX4, IncF, and IncP with the potential to mediate the dissemination of *mcr-1* genes into other gram-negative bacteria [28, 29]. In this study, *mcr-1* was associated with only one copy of IS*Apl1* in pEC012-1 while IS*Apl1* is most likely an important factor responsible for the insertion and fixation of the *mcr-1* gene into various classes of self-transmissible plasmids and host chromosomes [30]. There is increasing evidence that the *mcr-1* gene is mobilized primarily as a composite transposon, which is made up of two copies of IS*Apl1* that bracket cassettes [31]. Snesrud et al. [30] analysis of the *mcr-1* sequence environment showed that Tn6330 has a strong tendency to decay through deletion, removing parts of, or both copies of IS*Apl1*, thus transfixing *mcr-1* into a vector plasmid. The loss of IS*Apl1* elements results in the loss of transposability, stabilizing the *mcr-1* cassette in plasmids, which facilitates the widespread dissemination of the colistin resistance gene in self-transmissible plasmids. Snesrud et al. also suggested that the transposase encoded by the upstream IS*Apl1* can recognize the downstream inverted repeat right and thereafter still be able to mobilize the *mcr-1*-*pap2* region without a complete composite transposon [30].

The gene *bla*_CTX-M-14_ belongs to *bla*_CTX-M-9-group_, possessing superior hydrolytic activity against ceftriaxone and cefotaxime rather than ceftazidime. The coexistence of *mcr-1* and ESBL genes in MDR *E. coli* isolates was first reported in China in 2016 [32]. Other reports have demonstrated that ESBL-producing *E. coli* were more likely to carry *mcr-1* than non-ESBL-producing *E. coli* [5, 33].

Importantly, *mcr-1* coexisted with other 14 ARGs in IncHI2 plasmid. IncHI2 plasmids play an important role in the dissemination of ARGs conferring resistance to *β*-lactams, cephalosporins, aminoglycosides, sulfonamides, quinolones, macrolides, fosfomycin, chloramphenicol, and tetracycline among Gram-negative bacteria as our research shows [34]. These ARGs can further exacerbate the spread of colistin-resistant isolates among animals, humans, and environments [35]. In this study, segment organized as IS*1*-IS*6*-*tet(M*)-*tcpC*-Tn*3* and partial-MFS transporter-*mph(A*)-IS*26* was found in the backbone of IncHI2-type conjugative plasmid different from MG656414.1 and MW815279.1. IS*26* was indicated to be a reason for formation of *mcr-1*-bearing IncHI2/HI2A/N/FII/FIA hybrid plasmid [3]. The tetracycline resistance gene *tet(M*) encodes a ribosomal protection protein that confers tetracycline resistance to a variety of bacterial species. Although *tet(M*)-like genes are most commonly found in bacterial chromosome [36], they also exist in conjugative plasmids from bacteria of varies species. Tet(M) with multiple mutations have been reported to confer resistance to tigecycline in *Streptococcus suis* [37]. The IncHI2-type conjugative plasmids carrying *mcr-1* and other 14 ARGs found in this study may spread between and across genera in the future, threatening antimicrobials treatment in clinical practice.

In the conjugation experiment, the plasmid pEC012-5 can be transferred from EC012 isolate to the *E. coli* 600. The MIC results indicated that the *bla*_TEM-176_ can mediate high levels of resistance to third-generation cephalosporins. The *β*-lactamase gene *bla*_TEM-176_ which was first reported in an *E. coli* strain D7111 isolated from the feces of a child in Peru (GenBank accession no: GU550123). TEM-176 differs by only one amino acid from TEM-1 (A222V). Spontaneous mutations occur in the *bla*_TEM-1_ gene can lead to changes in enzyme activity causing resistance to third- or fourth-generation cephalosporins [38]. Nevertheless, since its first report in GenBank in 2010, *bla*_TEM-176_ has been reported in microorganisms isolated from samples of different origins (e.g. human, companion animals or wild animals), and from several geographic locations, such as Austria, Singapore, or Japan, thus showing worldwide dissemination [39–43]. Although few data are available on its genomic location, previous studies have shown its presence in IncX-group plasmids [44], the plasmid pEC012-5 also confirms this fact.

In this study, WGS analysis revealed that EC012 strain belonged to the ST295 lineages, respectively. In China, *E. coli* ST295 lineage has been detected only once from a diarrhea human sample in 2018. This study reports for the first time the *E. coli* ST295 lineage from poultry in China. The EC012 isolate was nearly to an isolate from human in the United Kingdom (Genbank accession no: SRR12541334), it indicated ST295 may be emerging as a pathogen in humans. Plasmids carried by *E. coli* ST295 play an important role in the carriage and dissemination of resistance to clinically important antimicrobials, particularly ESBL genes such as *bla*_CTX-M_, and possibly *mcr* genes. Besides, plasmid acquisition has played an important role in the evolution of *E. coli* ST295 [45].

Foodborne transmission is a possible route of transmission of *mcr*-positive *E. coli* from animals exposed to colistin [46]. A study found a positive correlation between *mcr-1* producing *E. coli* carriage in the human normal flora and the consumption of colistin-exposed farm animals [8]. This data highlights the presence of *mcr*-positive *E. coli* in food-producing animals and the transmission route of *mcr*-positive *E. coli* from colistin-exposed animals to humans, which impacts public health care.

## 5. Conclusion

In summary, this study revealed the genomic features of a MDR APEC strain EC012 belonging to epidemic serotype O1:H16 and ST295. The EC012 carried multiple ARGs, including *mcr-1*, *bla*_CTX-M-14_, and *bla*_TEM-176_, which existed in transferable plasmids and mediated the resistance to colistin and third-generation cephalosporins. The results indicated that APEC was a potential reservoir of colistin-resistance gene *mcr-1* and ESBL encoding genes. However, the detailed mechanisms and genetic diversity of these ARGs in different bacteria remain unclear. Thus, it is necessary to strengthen the surveillance of ARGs dissemination among bacteria from different origins.

## Figures and Tables

**Table 1:** MICs of antibiotics for EC012, *E. coli* 600, and transconjugates.

Antibiotics	EC012	*E. coli* 600	*E*. *coli* 600 with pEC012-1	*E*. *coli* 600 with pEC012-5
Cefotaxime	>64	<0.5	>64	>64
Cefuroxime	>32	1	>32	>32
Ceftriaxone	>128	<0.064	>128	>128
Ceftazidime	1	<0.5	4	8
Rifampicin	4	>16	>16	>16
Tetracycline	16	4	16	4
Tigecycline	0.25	0.25	0.125	0.125
Ciprofloxacin	>64	1	1	32
Polymyxin B	4	0.5	2	1
Imipenem	0.125	0.25	0.25	0.125
Meropenem	<0.016	<0.016	<0.016	<0.016

**Table 2:** Genome features and resistome of EC012.

Feature	Chromosome	pEC012-1	pEC012-2	pEC012-3	pEC012-4	pEC012-5	pEC012-6
Size (bp)	4,771,666	257,019	123,652	84,973	46,420	42,886	3,240
GC content (%)	50.76	46.46	49.01	48.37	52.46	45.73	43.58
ORF num	4,429	294	150	112	40	51	2
Inc group (pMLST)	NA	IncHI2/HI2A/N	IncFIA/N	IncFIB	IncFII	IncX1	ColRNAI
Resistome	—	—	—	—	—	—	—
Polymyxins	*—*	*mcr-1*	*—*	*—*	*—*	*—*	—
*β*-Lactams	*ampC*, *ampH*, and *ampC1*	*bla*_CTX-M-14_	*—*	*—*	*—*	*—*	—
Cephalosporins	*—*	*—*	*—*	*—*	*—*	*bla*_TEM-176_	—
Aminoglycosides	*—*	*aac(3)-IVa*, *aph(4)-Ia*, *aadA2*, *aadA1*, and *APH(3')-Ia*	*—*	*—*	*—*	*aph(3')-Ia*	—
Quinolones	*—*	*qacH*	*—*	*—*	*—*	*qnrS9 and* partical	—
Sulphonamides	*—*	*sul2 and sul3*	*—*	*—*	*—*	*—*	—
Macrolides	*—*	*mph(A)*	*—*	*—*	*—*	*—*	—
Fosfomicin	*—*	*fosA3*	*—*	*—*	*—*	*—*	—
Chloramphenicol	*—*	*floR and cmlA1*	*—*	*—*	*—*	*cmlA*	—
Tetracyline	*—*	*tet (M)*	*—*	*—*	*—*	*—*	—

## Data Availability

Datasets used and/or analyzed during this study can be obtained from the corresponding author on reasonable request. All sequencing data are available at NCBI: chr-EC012 (https://www.ncbi.nlm.nih.gov/nuccore/CP119577.1), pEC012-1 (https://www.ncbi.nlm.nih.gov/nuccore/CP119578.1), pEC012-2 (https://www.ncbi.nlm.nih.gov/nuccore/CP119579.2), pEC012-3 (https://www.ncbi.nlm.nih.gov/nuccore/CP119580.2), pEC012-4 (https://www.ncbi.nlm.nih.gov/nuccore/CP119581.2), pEC012-5 (https://www.ncbi.nlm.nih.gov/nuccore/CP119582.2), and pEC012-6 (https://www.ncbi.nlm.nih.gov/nuccore/CP119583.2).
